# Role of Biomaterials in the Development of Epithelial Support in 3D In Vitro Airway Epithelium Development: A Systematic Review

**DOI:** 10.3390/ijms241914935

**Published:** 2023-10-05

**Authors:** Ab Karim Nashihah, Fairuz Izan Muhammad Firdaus, Mh. Busra Fauzi, Nadhratun Naiim Mobarak, Yogeswaran Lokanathan

**Affiliations:** 1 Centre for Tissue Engineering and Regenerative Medicine, Faculty of Medicine, Universiti Kebangsaan Malaysia, Kuala Lumpur 56000, Malaysiafirdaus.dann@gmail.com (F.I.M.F.); fauzibusra@ukm.edu.my (M.B.F.); 2 School of Chemical Sciences and Food Technology, Faculty of Science and Technology, Universiti Kebangsaan Malaysia UKM, Bangi 43600, Malaysia; nadhratunnaiim@ukm.edu.my

**Keywords:** airway epithelium, biomaterial, scaffold, mucociliary differentiation, in vitro

## Abstract

Respiratory diseases have a major impact on global health. The airway epithelium, which acts as a frontline defence, is one of the most common targets for inhaled allergens, irritants, or micro-organisms to enter the respiratory system. In the tissue engineering field, biomaterials play a crucial role. Due to the continuing high impact of respiratory diseases on society and the emergence of new respiratory viruses, in vitro airway epithelial models with high microphysiological similarities that are also easily adjustable to replicate disease models are urgently needed to better understand those diseases. Thus, the development of biomaterial scaffolds for the airway epithelium is important due to their function as a cell-support device in which cells are seeded in vitro and then are encouraged to lay down a matrix to form the foundations of a tissue for transplantation. Studies conducted in in vitro models are necessary because they accelerate the development of new treatments. Moreover, in comparatively controlled conditions, in vitro models allow for the stimulation of complex interactions between cells, scaffolds, and growth factors. Based on recent studies, the biomaterial scaffolds that have been tested in in vitro models appear to be viable options for repairing the airway epithelium and avoiding any complications. This review discusses the role of biomaterial scaffolds in in vitro airway epithelium models. The effects of scaffold, physicochemical, and mechanical properties in recent studies were also discussed.

## 1. Introduction

Respiratory diseases have a massive impact on global health. The top 20 causes of disability-adjusted life years (DALYs) in the 2020 Global Burden of Disease Report include four of the “big five” lung diseases after examining all ages and all nation income levels [1]. The fourth major cause is lower tract respiratory infection, the sixth is chronic obstructive pulmonary disease (COPD), the twelfth is tuberculosis (TB), and the seventeenth is lung cancer. According to the Forum of International Respiratory Societies held in 2017, there are an estimated 65 million people with moderate to severe COPD, about 334 million people suffer from asthma, 10.4 million people developed TB in 2015, acute lower respiratory tract infections have been identified as one of the top three causes of death and disability in both children and adults, and lung cancer kills 1.6 million people each year. In addition, inhalation of particulate matter (PM) has been linked to morbidity and mortality in cardiopulmonary disorders such as COPD, asthma, lung cancer, pneumonia, ischemic heart disease, and stroke in recent epidemiological and experimental studies [2,3]. The nose, as the primary organ exposed to environmental microbes, pollutants, and allergens, plays a leading role in the development of respiratory diseases. Therefore, it is not surprising that upper airway inflammation is so common nowadays. According to one researcher [4], epithelial cells, which form a protective lining on the surfaces of airways and alveoli within the lungs, serve as the initial recipients of inhaled substances originating from various external sources, such as the environment or workplace. Following an injury, the airway epithelium must undergo rapid repair and renewal to regain its integrity and functionality [5].

The airway epithelium plays an important role in the lungs’ defence against pathogens. In vitro models can be used to investigate the basic steps of the wound healing process, as well as the role of various cytokines and extracellular matrix (ECM) components in triggering behavioural changes in wound-healing epithelial cells. Moreover, the airway epithelium maintains a conduit for air to pass to and from the alveoli. Airway epithelium cells (ECs) line the airways and transfer gases to and from the alveoli [4]. They are the most numerous cell type in the lung and the first to meet inhaled chemicals; hence, they play a crucial role in host defence control. The airway tract can be divided into two zones: the respiratory zone, responsible for oxygenating the blood, and the conditioning zone, which cleans, moistens, and transports inhaled air to the distal part of the airways [6]. In addition, the continuous layer that covers the respiratory tract’s surface known as the airway epithelium is made up of cells joined by adhesion and tight junctions [7]. In the proximal intrapulmonary airways, the epithelium is pseudostratified, with basal cells serving as progenitors [8].

Moreover, the airway epithelium, which lines the respiratory tract, possesses several key features that enable it to perform crucial functions in the respiratory system. Some of the main features of the airway epithelium include ciliated and mucus-producing cells. Both types of cell beat in coordinated motions to move mucus and trapped particles upward, helping to clear the airways of debris and pathogens [9]. Goblet cells, interspersed within the epithelium, produce mucus, further aiding in trapping and expelling inhaled contaminants [10], while the tight junctions between epithelial cells form a barrier, regulating ion and molecule passage while safeguarding the airway’s integrity [11]. Additionally, immune cells, including macrophages and dendritic cells, are stationed within the epithelium to detect and respond to pathogens [12]. The airway epithelium’s capacity for self-repair and regeneration ensures resilience in the face of damage, while sensory receptors allow it to detect irritants and initiate protective measures. These remarkable features collectively enable the airway epithelium to serve as a critical defence mechanism against respiratory infections and maintain the vital process of gas exchange in the lungs.

To ensure adequate health and safety conditions, it is increasingly essential to establish credible experimental models for the clinical utilization of biomaterials and for predicting the success or failure of implants. The practice of operating outside of a living creature in a controlled environment is known as in vitro modelling. In vitro models have quickly gained the potential to establish a link between animal and clinical studies by providing more comprehensive systems that mimic the natural key aspects of human tissue structure, biochemistry, biomechanics, and functions [13]. Moreover, a recent study successfully proved that in vitro human alveolar models bring a great new powerful platform for discovering the cellular behaviour and activity of human lung alveolar stem/progenitor cells, cell–cell crosstalk, host–pathogen interactions, drug screening, and toxicity studies [14].

Biomaterials provide artificial tissues with physical support as well as potent topographical and chemical cues to guide cell behaviour. The fabrication of biomaterial scaffolds in an in vitro airway epithelium model may be a good solution to assist in the formation of new functional tissues for medical purposes. Scaffolds act as three-dimensional porous solid biomaterials to provide a template for the regeneration of defects while encouraging cell attachment, proliferation, extracellular matrix production, and the restoration of arteries, nerves, muscles, and bones, among other things [15]. Different biomaterial scaffolds can be tuned to adjust porosity, gelation time, and degradation rate to provide tailorable biomaterials that potentiate the cell survival and differentiation that enable therapeutic effects. Moreover, to improve cell signalling or ECM production, the biomimicry methodology of arranging diverse cellular components to mimic living tissue like growth factors, hormones, ECM proteins, and others has been applied [16].

To this point, there has been no comprehensive systematic review or meta-analysis conducted to explore the role of biomaterials in the development of the airway epithelium in in vitro models. Given the current high level of interest in biomaterial-based interventions, it is now opportune to undertake such a review. Our systematic review and meta-analysis have the overarching goal of evaluating the potential of appropriate biomaterials for constructing an in vitro model of airway epithelium development. This review delves into the pivotal role played by biomaterial scaffolds within the context of in vitro models of the airway epithelium. It explores how these scaffolds contribute to the development and functionality of these models. In addition to discussing the general significance of biomaterial scaffolds, this review takes a closer look at the specific effects they exert. This examination encompasses the scaffold’s physicochemical properties, such as its composition, surface characteristics, and chemical interactions, as well as its mechanical properties, such as stiffness and elasticity. Recent studies investigating these aspects are thoroughly examined and analysed. By doing so, this review aims to provide a comprehensive understanding of how biomaterial scaffolds influence the creation and performance of in vitro airway epithelium models, shedding light on their potential applications and advancements in the field. Thus, this review is important to understand the properties of ideal biomaterials for an in vitro airway epithelium model and to enhance the development of suitable biomaterials in the future for use as part of an in vitro airway epithelium model.

**Figure 1:**
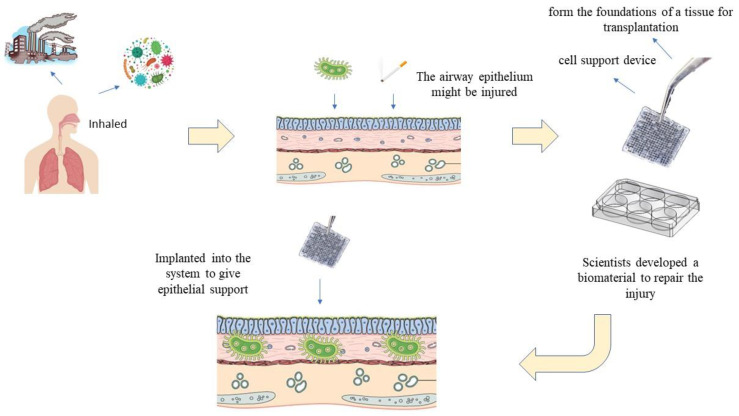
The use of ideal biomaterial for in vitro airway epithelium model.

Figure 1 shows the use of an ideal biomaterial for the in vitro airway epithelium model.

## 2. Materials and Methods

### 2.1. Search Criteria

Comprehensive research articles were retrieved from three databases: PubMed, Scopus, and Science Direct. The Preferred Reporting Items for Systematic Review and Meta-Analyses (PRISMA) guidelines were used to identify and retrieve the literature [17]. The search strategy or searching method used two sets of keyword combinations, which were “biomaterials” OR “scaffold” OR “material” OR “natural material” AND “airway epithelium” OR “respiratory epithelium” AND “in vitro”. Any disagreements between the authors were settled by reviewing the abstracts and full texts to ascertain the criteria employed in the studies. Table 1 shows the full list of the inclusion/exclusion criteria imposed.

### 2.2. Data Extraction and Analysis

Specific categories for data analysis were developed before evaluating the recognized publications; these related to the cellular and biomaterial inputs that were used in the airway epithelium and the biological and biomechanical outcome measures. The cellular input comprised the type of cells used while biomaterial inputs comprised biomaterial composition, technique of further crosslinking, and biological functionalization. In this analysis, three steps were used to fulfil the requirements of this review. First, the titles of the articles were screened. Then, the selected papers were subjected to an abstract analysis. Lastly, articles published that did not focus on the specific categories stated above were removed.

## 3. Results

### 3.1. Study Characteristics

**Figure 2:**
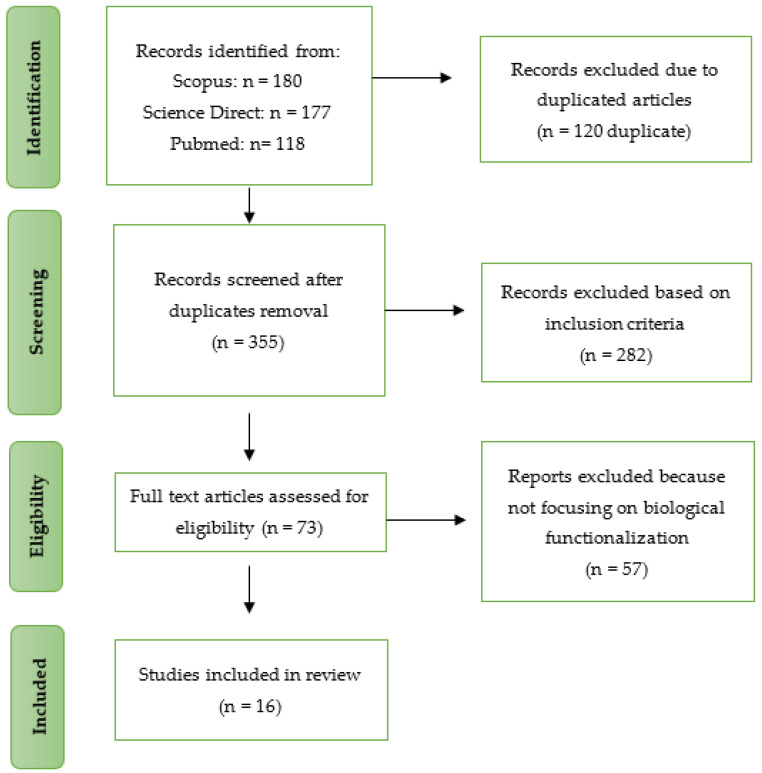
Flow diagram of article selection and data extraction management.

Among the articles chosen, all the articles were biomaterials-based and reported on physicochemical, mechanical, and biological properties, particularly in tissue engineering and regenerative medicine. All of the studies were published between the years 2012 and 2022. The flowchart in Figure 2 summarizes the study selection approach. With that, the data were summarized including physicochemical and mechanical properties and biological properties such as cellular response. A summary of all the studies included is displayed in Table 2.

### 3.2. Physicochemical, Mechanical, and Biological Properties

In developing a new biomaterial for future use in an in vitro airway model, there are physicochemical and mechanical properties such as biodegradation, swelling ratio, porosity, ultrastructure, mechanical strength, contact angle, and water vapor transmission rate (WVTR) that need to be studied. Mechanical properties, corrosion/degradation resistance, and electrical/optical properties are the most important physicochemical features of a material [18]. In this review, 8 out of the 16 articles reported their findings on the physicochemical and mechanical properties of the biomaterials or scaffolds, including ultrastructure, porosity, contact angle, degradation, and trans-epithelial electrical resistance measurements, while the other articles focused on the biological properties. For ultrastructure, the average diameter of the scaffolds is between 0.24 and 454 µm [18,19,20], while for pore and porosity, the average of the data is between 2.8 and 469 µm and 10.45 and 90% [20,21,22]. According to one researcher [19], the contact angle of their Electrospun Poly (Methylmethacrylate) (PMMA) nanofibers was higher than 90°, while in the study reported in [20], their hydrogel showed that the contact angle was lower than 90°, which indicated hydrophilicity of the scaffolds.

Cell attachment and spreading are two of the important biological properties to observe in the development of suitable biomaterial scaffolds. According to several recent studies, the structural characteristics of a scaffold control the biological qualities of cells, which are essential for the formation of tissue substitutes [23]. In addition, cell attachment is the first stage in a series of cell–biomaterial interactions, and it is critical for cellular activities like cell guidance, proliferation, and differentiation. Cell attachment assays are used to measure adhesion between cells or between a cell and a surface or extracellular matrix. The objective of this parameter is to determine the percentage of cell attachment at a certain time after the cell was seeded. The cell attachment rate needs to be higher than 80% to achieve a good result in cell attachment and spreading. Epithelial cells must attach and form a monolayer to achieve epithelial barrier function [24]. In this review, in vitro outcomes including cell attachment, differentiation, and proliferation were also measured. The most used cell types in the studies are human bronchial epithelial cells (HBECs), respiratory epithelial cells (RECs), and the bronchial epithelial cell line (Calu-3). The results are summarized in Table 2.

**Table 2:** A summary of studies of physicochemical, mechanical, and biological properties of biomaterials/scaffolds in in vitro airway epithelium models.

Type of Biomaterials/Scaffolds	Type of Cells	Outcomes	Conclusion
Microgrooved gelatin hydrogel crosslinked with glutaraldehyde [25]	Human bronchial epithelial cell line (BEAS-2B)	**1. Topographical** The diffraction grating had a pitch of 1 μm and 186 nm depth. Optimized using 5% gelatin and 1% glutaraldehyde. Average shrinkage during the freeze–drying process in macroscopic gel dimensions: - Gels with shallow grooves: 2.3 ± 0.1 times and 2.4 ± 0.2 times. - Gels with deep grooves: 1.1 ± 0.02 times and 1.1 ± 0.1 times. **2. Cell attachment, spreading, and metabolic activity** Allowed 99.48 ± 0.52% of BEAS-2B spreading over 10 days. Increasing BEAS-2B metabolic activity over 10 days. BEAS2B cell confluent cultures spread randomly on flat PDMS and gelatin. Cells aligned in the direction of the microgrooves on the hydrogel. The number of cilia generated was not significantly different for the gelatin inserts compared to standard transwell.	Topographical cues can control the organization and apicobasal polarization of epithelial cell lines. Potentially helpful for examining the connection between the tissue structure and function of primary cell and cell alignment.
Non-woven bilayered biodegradable chitosan-gelatin-polylactide (CGP) with hyaluronic acid (HA) immobilization scaffold [22]	Human respiratory epithelium cells (HRECs)	**1. Physicochemical properties** Electrospun nanofibrous layer - Pore size: 2.8 ± 0.1 μm - Layer thickness: 53 ± 5 μm - Fibre diameter: 0.35 ± 0.2 μm - Porosity: 90 ± 3% - Contact angle: 114° Electrospun microfibrous layer - Pore size: 37.5 ± 3.8 μm - Layer thickness: 180 ± 3 μm - Fibre diameter: 2.5 ± 2.4 μm - Porosity: 88 ± 3% - Contact angle: Not stated **2. Cellular Responses** Cell proliferation: the HRECs actively migrate, proliferate, and reach confluence on the surface of the nanofibrous layer. Cell differentiation: regardless of the HA treatment, boosting the overall scaffold thickness to 350 µm led to the inhibition of mucociliary differentiation and the development of squamous epithelium.	Epithelial cell development is supported by the upper (nanofibrous) layer, which also serves to stop cell migration into the scaffold’s interior. The lower microfibrous layer was developed to allow fibroblasts to migrate inside and occupy the entire volume of the layer uniformly. The scaffold was immobilized using HA to increase the hydrophilicity of the material.
Three-dimensionally printed porous structure of a thermoresponsive injectable polyethercarbonate (3D-TIPS) stiffness-softening elastomer nanohybrid impregnated with collagen nanofibrous hydrogel [26]	Human bronchial epithelial cells (hBEpiCs) Human bronchial fibroblast cells (hBFs) Human bone marrow mesenchymal stem cells (hBM-MSCs)	**1. Mechanical properties** When collagen hydrogel was added to the 3D-TIPS scaffold, the strength and modulus remained the same. Following a 28-day isothermal relaxation period at 37 °C, both the tensile and compression modulus decreased. During the stiffness-damping effect, the scaffold grew softer as reversible compliance increased. **2. Morphology** Collagen hydrogel coating created an additional nanofibrous structure that permeated the interconnected printed network The hybrid hydrogel more closely resembled the structure and function of the trachea’s extracellular matrix. **3. Metabolic activity and proliferation** For 10 days, HBEpiCs cultured on collagen hydrogel-functionalized composite scaffolds (3D-TIPS + Collagen) demonstrated noticeably higher metabolic activity and proliferation than the untreated 3D-TIPS scaffolds (*p* < 0.001). **4. Immunocytochemistry** The majority of hBEpiCs still had basal cells that were not differentiated. When hBM-MSCs were cocultured with hBEpiCs on 3D-TIPS + Collagen scaffolds, the proportion of labeled mitotic cells was lower (Ki-67). Improved lamellipodia generation on scaffolds when collagen hydrogel hBFs and hBM-MSCs were cocultured.	3D-TIPS+Collagen hydrogel can support the growth and differentiation of the human bronchial epithelium. The maturation of the epithelium accelerated when the hybrid hydrogel coculture with hBM-MSCs was used.
Electrospun nanofibers of poly(ε-caprolactone)/ depolymerized chitosan (PCL/chitosan) [27]	Porcine tracheobronchial epithelial (PTBE) cells	**1. Mechanical properties** PCL nanofibers alone have > elasticity past 200% of the original gauge length. PCL nanofibers by themselves had a noticeably higher percentage elongation break, demonstrating their well-known elasticity. Y modulus - PCL/Chitosan 100: 9 ± 1.3 MPA - PCL/Chitosan 90/10: 8 ± 1.8 0.6 MPA - PCL/Chitosan 80/20: 16 ± 1.6 MPA **2. Fiber morphology** Higher PCL/chitosan ratio solutions demonstrated greater electrospinnability with fewer beads and more smooth fibers. **3. Structural of fiber** FTIR reading in PCL: characteristic carbonyl (C=O) peak at 1720 cm^−1^, CH_2_ stretching at 2950 cm^−1^ (asymmetric) and 2865 cm^−1^ (symmetric), C-O stretching at 1050 cm^−1^, and C-O-C stretching at 1240 cm^−1^. **4. Cytotoxicity Analysis** For all of the PCL/chitosan nanofibers, LDH levels were essentially the same. The outcome demonstrates neither the PCL/chitosan material’s topology nor its ability to cause more cell damage or death. **5. Cell Morphology Observation** PTBE cells adhered well to the topology of the nanofibers and dispersed throughout it, forming cell clusters. The PCL/chitosan nanofibers show well-integrated PTBE cells with a diameter of 8–10 m. In 100/0 and 90/10 fibers, cell bodies could be plainly observed. There was an expansion of cell bodies in the 80/20 and 70/30 nanofiber membranes.	The PCL/chitosan nanofibers displayed good homogeneity, structural integrity, appropriate mechanical characteristics, and cellular compatibility. The scaffold can support the growth of PTBE.
Electrospun poly (methylmethacrylate) (PMMA) nanofibers Collagen-coated PMMA nanofibers (PMMACOL) [19]	Respiratory epithelial cells (RECs)	**1. Contact angle** PMMA nanofibers (PMMA) at 0 h before UV irradiation: 131.89° ± 1.33°. PMMA after 6 h of UV irradiation: 110.04° ± 0.27° (*p* < 0.05). Genipin-crosslinked collagen-coated PMMA nanofibers (PMMAGEN): 108.37° ± 1.4 °. **2. Ultrastructure** The average diameter of the randomly aligned PMMA nanofibers: 298.44 ± 17.33 nm. The collagen-coated nanofibers were visible and showed the collagen had established networks between the neighboring fibers in collagen-coated PMMA nanofibers (PMMACOL), UV-irradiated collagen-coated PMMA nanofibers (PMMAUV), and PMMAGEN. The PMMACOL and PMMAUV nanofibers’ collagen-coated surfaces had a flattened shape and rough surface features. PMMA nanofibers have flat surfaces without a collagen coating. More collagen was deposited around and between the fibers in PMMAGEN. **3. Cell Morphology, Attachment, and Proliferation** On tissue culture plates, RECs grow as colonies with polygonal forms throughout. Day 4: in comparison to PMMACOL and PMMAUV, PMMAGEN and PMMA had lower cell attachment Day 9: on PMMAUV, the highest REC counts were recorded (6.44 × 10^4^ ± 2.77 × 10^4^ cells/cm^2^). PMMAUV has the highest REC proliferation rate (0.005 ± 0.003 h^−1^).	The ability of the collagen to adhere to the PMMA sheet is improved by genipin crosslinking in comparison to UV radiation and the conventional collagen-coating process. The PMMA group exposed to UV light produced the highest rate of REC proliferation and growth while retaining REC features. PMMA nanofiber sheets electrospun with UV radiation were chosen to build an in vitro RE model.
Three-dimensional-printing of silk fibroin/hydroxypropyl methylcellulose (SF/HPMC) thixotropic hydrogel [20]	Normal human bronchial epithelial cell line (BEAS-2B)	**1. Ultrastructure** Mean diameter of the single-printed bar: 454 ± 7 µm Smooth surface **2. Pore size** Macropore diameter: 469 ± 19 µm Macropores with micropores visible in their sidewalls. The scaffolds’ cross-section revealed that the printed bar had a tiny-pore structure. **3. Porosity** The printed SF/HPMC scaffold-f had relatively higher porosity (58.39 ± 1.25%). **4. Contact Angle** The contact angle of the droplet was <90° and the scaffolds possessed good hydrophilicity. **5. Mechanical Properties** SF/HPMC scaffolds-f with a thickness of 1.8 mm (0.17 ± 0.02 MPa). **6. Cytoxicity test** Cell viability of higher than 90%. Cell viability and SF/HPMC concentration both increased. The biocompatibility of SF was minimally affected by the addition of HPMC. No potentially dangerous chemical agent was used. **7. Cell proliferation** 1, 3, 5, and 7 days: the cell-seeded SF/HPMC scaffold-f assay’s OD value increased. During the culture phase, BEAS-2B cells were able to proliferate on the 3D-printed SF/HPMC scaffolds. **8. Cells morphology and viability** Day 1: on the surfaces, the cells were scattered and isolated. BEAS-2B cells appeared to attach to one another and form cellular aggregates on the wall of macropores. BEAS-2B cells demonstrated strong adhesion, proliferation, and growth on the scaffolds’ cross sections. Day 7: the cells attached to the scaffolds were alive and continued to proliferate.	The 3D SF/HPMC scaffold-f produced via 3D printing SF/HPMC thixotropic hydrogel may promote in vitro the proliferation of tracheal epithelium. The scaffolds’ favorable mechanical and porosity characteristics facilitated the development of BEAS-2B cells.
Electrospun polyethylene terephthalate scaffold (PET) [18]	The epithelial cell line (Calu-3) The fibroblast (MRC-5) cell lines	**1. Trans-epithelial electrical resistance (TEER) measurements** TEER after 14 days in culture epithelial cells (cultured alone): ∼130 Ω cm^2^. Cells attained measurements of ∼200 Ω cm^2^ when cocultures of epithelial and fibroblast cells were used. **2. Ultrastructure** The mean fiber diameter of the PET scaffold: 240 ± 70 nm. The average thickness of the PET scaffold: 60 ± 10 μm. **3. Immunocytochemical** After 14 days: collagen and fibronectin, which are mostly prevalent in epithelial and fibroblast cells, as well as Ki67, were detected. **4.H&E staining** After 14 days at ALI, single cultures produced a single thin layer of cells, whereas cocultured epithelial cells showed a more layered and dense structure. Cocultures of epithelial cells showed an earlier mucus production than single cultures.	In comparison to single cultures, epithelial cells regenerated more quickly in cocultures after being exposed to chemical insult (i.e., an enzymatically active allergen). Cell migration and cell–cell interaction are possible in this 3D lung model. In this study, the 3D immunocompetent lung model presented successfully supports the culture of multiple cell types on electrospun PET scaffolds.
Novel electrospun biphasic scaffold [21]	MRC5 and CALU3 cell lines	**1. Diameter** Nanofiber: 0.25 ± 0.002 μm Microfiber: 2.5 ± 0.02 μm **2. Pore size** Nanofiber: 1.4 ± 0.01 μm Microfiber: 89.0 ± 0.4 μm **3. Porosity** Nanofiber: 84.3 ± 2.1% Microfiber: 10.45 ± 0.12% **4. Proliferation rate** Over 14 days: during the culture time, fibroblasts grown on the microfibre scaffold had a much higher viability than those grown on the nanofibre scaffold and were also noticeably longer than those grown on the nanofibre scaffold.	The pore size increased ten times with the increase in fiber diameter. Compared to cell interactions with several fibers on the nanofibre scaffold, fibroblast cell interaction with individual microfibres offers an environment for improved adhesion, viability, and for a more fibroblast-like elongated shape. In the development of a disease in a 3D environment, this scaffold might be loaded with entirely differentiated adult lung cells from people with airway diseases to examine significant intercellular interactions.
Collagen IV- and laminin-containing extracellular matrix [28]	Human bronchial epithelial cells (HBECs)	**1. Cell attachment, differentiation, and proliferation** Support cell attachment. No significant change in HBEC expansion. Cells failed to differentiate appropriately.	Collagen IV and laminin, two extracellular matrix proteins, are crucial for respiratory epithelial adhesion and growth in vitro.
Fibrin gel [29]	Respiratory epithelial cells	**1. Cell differentiation** Increase in dedifferentiated cells. Respiratory epithelial cells grown on pure fibrin gel. **2. Immunohistochemical staining** Pulmonary epithelial cells grown on a substrate containing fibroblasts experienced improved epithelial differentiation. Demonstrates the presence of claudin-1. Cells grown on pure fibrin. Gels eventually stop serving as barriers.	The amount of fibroblasts in the respiratory epithelium increased. The development of the respiratory epithelium is positively influenced by the addition of fibroblasts to fibrin gel.
Porous three-dimensional silk fibroin scaffolds (3D SF) [30]	Human tracheobronchial epithelial cells (HBECs)	**1. Cell viability and proliferation:** In the scaffold, the SEM result showed that HBECs demonstrated good adhesion, proliferation, and growth. At day 21, the cell junction became loose and the cell count dropped. It maintained strong viability over the entire 21-day process	For tracheal reconstruction, the SF scaffold is anticipated to be a promising replacement. The high-porosity 3D SF scaffolds aided in the development of HBECs.
Nanofibrous polycaprolactone–chitosan (PCL-chitosan) Scaffold All-trans retinoic acid (atRA)-loaded Scaffold [31]	Calu-3 bronchial epithelial cell line	**1. Biocompatibility:** The PCL and PCL-chitosan scaffolds are biocompatible and maintain Calu-3 cell viability for 14 days. The biocompatible substrates for respiratory epithelial cell development at RA-loaded scaffolds call for additional in vitro evaluation of epithelial behavior.	This scaffold integrates a synthetic polymer that has been used in human tracheal stents and a drug with decades of use in patients.
Microfluidic lung airway-on-a-chip with arrayable suspended gels [32]	Human airway epithelial cells (Calu-3 cell line) Human bronchial smooth muscle cells (hBSMCs)	**1. Cell adhesion:** For a 7-day culture period, the combination of Col-I and a high concentration of Matrigel allowed greater initial and sustained Calu-3 cell and hBSMC adherence. **2. ALI Culture** Each epithelial cell’s periphery had robust localization upon F-actin staining, which is consistent with a typical cell cortex seen with cells arranged in a monolayer. On the monolayer, distributed Calu-3 cell groups showed positive MUC5AC expression. After 31 days, when costained with the culture for ZO-I, a similar tight-junction formation was discovered.	ALI culture conditions in this study could be accommodated to promote goblet cell development in airway epithelial cells. Potential for use as a lung airway tissue model that can incorporate other microenvironmental cues. This system leads to the differentiation of airway epithelial cells into mucus-producing goblet cells inside the microfluidic device.
Human plasma [33]	Human tissue respiratory epithelial construct (HTREC) Respiratory epithelial cells (RECs)	**1. Histological analysis:** Day 4: the RECs’ cell count increased by day 4 after their incorporation. **2. Immunocytochemical:** Day 4: post-REC incorporation to the construct saw an increase in the number of Ki67-expressing cells compared to day 1 post REC inclusion. The RECs that were present within the human tissue respiratory epithelial construct were actively growing and multiplying (HTREC). **3. Gene expression analysis** RT-PCR: at day 4 of incorporating the RECs into the CaCl2-polymerised human plasma, the expression of the Ki67 gene had been significantly (*p* < 0.05) upregulated. **4. Profile of Respiratory Epithelial Cell Population within HTREC** The percentage of MUC5AC-positive cells when incorporating RECs within CaCl2-polymerised human plasma: - On day 1: 32.1% ± 3.56 - At day 4: 44.3% ± 4.53 The percentage of proliferative cells (Ki67-positive cells) post incorporating RECs within CaCl2-polymerised human plasma: - On day 1: 26.4% ± 3.39 - At day 4: 50.3% ± 6.92	This study successfully isolated RECs from nasal turbinate with their phenotype resembling native ones. The scaffold comprising human blood plasma polymerized with calcium chloride proves that the construct is supportive of the cell proliferation and mucin secretion phenotype of RECs. HTREC can be a suitable candidate for respiratory epithelial tissue reconstruction.
Urinary bladder-derived ECM hydrogels [34]	Human bronchial epithelial cells (HBECs)	**1. Cell Proliferation and differentiation** Day 4: on both ECM substrates, a confluent monolayer of cells developed. Day 19: similar to the natural airway epithelium, cells on the tracheal hydrogel displayed a pseudostratified columnar shape. When grown on the trachea and UBM hydrogel, HBEC had positive Alcian blue staining.	Urinary bladder-derived ECM hydrogels support the growth and differentiation of HBECs. The scaffold promotes a mature respiratory epithelium for regenerative medicine applications in the trachea.
Fibrin gels (FIB0.5) Agarose-type I collagen hydrogels (AGR0.5COLL0.5) [35]	Human umbilical venous endothelial cells (HUVECs) Human respiratory epithelial cells (HRECs) Human nasal fibroblasts (HNFs) Human dermal fibroblasts (HDFs)	**1. Cell differentiation and proliferation** The development of cilia and proliferation and differentiation are all positively impacted by FIB0.5 samples. The AGR0.5COLL0.5 group appears to be incapable of promoting epithelial differentiation. A confluent cell layer without the normal respiratory epithelial structure was visible in the epithelium on AGR0.5COLL0.5. Tight connections, goblet cells, and mucin secretion were not expressed in the confluent cell layer of the epithelial cells on AGR0.5COLL0.5. **2. Vascularization** **HDFs** - The volume of capillary-like structures in cocultures: 809,449 μm^3^ - The surface area of vascular structures: 335,040 μm^2^ - The length: 11,035 μm - Number of branches: 29 **HNFs** - The volume of capillary-like structures in cocultures: 738,448 μm^3^ - The surface area of vascular structures: 300,459 μm^2^ - The length: 9728 μm - Number of branches: 22 **3. Elastic Shear Modulus G’** AGR0.5COLL0.5: (5.0 ± 0.6); FIB0.5 (1.5 ± 0.53)	This study demonstrated that the addition of tracheal fibroblasts to fibrin gels improved HREC development and established the best possible framework for airway tissue engineering. HNFs can support the differentiation of the cells and showed no discernible difference in vascularization-supportive capability. The study successfully tri-cultivated HRECs forming a pseudostratified and ciliated epithelium with HUVECs and fibroblasts revealing vascularization within fibrin gel.

## 4. Discussion

In tissue engineering, approaches to the development of biomaterials in airway regeneration, especially the airway epithelium, are currently limited. Currently, in vitro models with high microphysiological similarity, that are at the same time easily tuneable to simulate disease models, are not readily available due to a lack of knowledge on the topographical and biochemical requirements for the formation of an in vitro airway epithelium with mucociliary function. Therefore, this review was conducted to determine the best biomaterials to develop an airway epithelial graft for future use. According to a study [24], there are selection criteria to identify the potential biomaterial based on specific criteria, for example, the epithelial graft must have a good mechanical strength, of more than 1.39 ± 0.16 MPa, to allow for transferability and surgical manipulation. Other than that, the biomaterials must be maintained from degradation until epithelial development is complete. Cell attachment and spreading also need to achieve at least 80%, the metabolic activity must be maintained, early focal adhesion formation is required within 2 h, and differentiation into 20% ciliated cells and 5% goblet cells is necessary.

Based on a previous study, mechanical properties are those that influence a material’s mechanical strength and ability to be moulded into a desired shape [36]. Moreover, some treatments might be affected by the mechanical properties of the material such as the heat treatment procedure and the operating temperature, which can alter the mechanical properties. There are numerous experimental methods for the characterization of biomaterials that are extensively used such as tensile yield stress, Young’s modulus, and fatigue stress. For the best mechanical properties, the biomaterial scaffold used in the airway epithelium must have mechanical strength that allows for transferability and surgical manipulation; as an example from a recent study, it was found that the PCL/chitosan scaffold had great mechanical strength compared to others [27].

We discovered that the choice of cell type, the type of materials to fabricate the biomaterial, and the study duration varied greatly between studies and that these differences may have influenced the studies’ outcomes in many cases. Furthermore, certain constraints of the cell culture used for implantation on the biomaterials were sometimes limited, such as the expenses of cell procurement, possible immunogenic responses, and regulatory hurdles placed on biological devices [37]. From a previous study, the urinary bladder-derived ECM hydrogels can support the growth and differentiation of HBECs [34]. Moreover, they also demonstrated excellent homogeneity, structural integrity, and cellular compatibility. In addition, when using a cell-seeded biomaterial method, the pre-implantation culture period is more important than cell type [38]. In this review, we found that the microgrooved gelatin hydrogel crosslinked with glutaraldehyde allowed 99.48% of BEAS-2B spreading by 10 days, while for the metabolic activity increased over 10 days [25]. Other than that, HRECs actively migrated, proliferated, and reached confluence on the nonwoven bilayered biodegradable chitosan–gelatine–polylactide (CGP) with immobilised hyaluronic acid (HA) scaffold but, unfortunately, the mucociliary differentiation was inhibited when the overall scaffold thickness was increased to 350 µm, regardless of HA treatment [22]. In addition, one study found that the metabolic activity and proliferation after 10 days were higher in 3D-TIPS with collagen compared to untreated 3D-TIPS scaffolds, and mostly hBEpiCs that still contained basal cells were not differentiated [26].

Furthermore, a cell viability test must be conducted to understand the conditions and also limits related to biocompatibility. The importance of running the test is that it can give valuable insight into a variety of elements of the material or structure, including the acceptability of surface changes, three-dimensional architecture, oxygen transport, compatibility with degradation products, and many more [39]. In this review, there are a few studies that conducted cell viability tests. A previous study proved that the cell viability on the scaffold was higher than 90% and the addition of HPMC may not be affected by the biocompatibility of SF; the HBECs showed good adhesion, proliferation, and growth and could maintain strong viability on the scaffold over 21 days, while the PCL and PCL-chitosan scaffolds were biocompatible and could maintain cell viability for 14 days [20,30,31]. For cell proliferation, the BEAS-2B cells were able to proliferate on the 3D-printed scaffold.In SF/HPMC scaffolds during the culture phase, the BEAS-2B cells demonstrated strong adhesion, proliferation, and growth on the scaffolds [20].

Biodegradable scaffold design is fundamental to the advancement of tissue engineering and organ regeneration. The rapid biodegradation of biomaterials after implantation is currently a major flaw in tissue engineering products [40]. Moreover, the reason that biodegradable scaffolds are widely regarded as essential components in tissue engineering is because they serve as temporary templates with mechanical and biological properties that are similar to those of the natural extracellular matrix (ECM) [41]. Before the regeneration of biologically functional tissue or a natural ECM, the biodegradable scaffold enables the manipulation of cell adhesion, invasion, proliferation, and differentiation. However, after a certain period, the scaffold is no longer needed and should be deteriorated [42]. With hydrolysable polymers, this may be achievable, resulting in nontoxic degradation products. Based on this review, only one study reported the degradation of a scaffold. The gelatine hydrogel reported by one researcher [25] could only maintain 29.900 ± 5.096% of its initial mass in lysozyme, while it degraded completely in all other enzyme combinations within the first week.

In addition, in airway epithelium, the mucociliary clearance function is an important basic innate defensive mechanism that protects the lungs from pollutants, allergens, and infections absorbed through the airway. The mucociliary escalator is controlled by respiratory cilia, which operate in conjunction with secreted airway mucus to remove inhaled debris and pathogens from the conducting airways [43]. Additionally, cilia in the lungs are also one of the initial points of contact between the host and inhaled micro-organisms. A multitude of respiratory conditions, such as COPD, cystic fibrosis, sinusitis, and persistent respiratory infections, are associated with impaired mucociliary function caused by abnormal cilia [44]. Most of the biomaterials or scaffolds in this review showed that they could support cell differentiation, except for the collagen IV- and laminin-containing extracellular matrixes and the AGR0.5COLL0.5 scaffold, in which the cells in the scaffold failed to differentiate properly [35,45].

Moreover, when contemplating the selection of biomaterials for forthcoming research, it is essential to meticulously assess a range of crucial factors. The top priority among these is biocompatibility, which dictates how effectively the material interacts with living tissues and whether it triggers any adverse responses. The mechanical attributes of the biomaterial are of great significance and guarantee that it possesses the necessary strength and durability for its intended purpose. Other than that, biodegradability is another factor of importance, particularly when applications require gradual absorption. Careful examination of the chemical composition is imperative to avoid potential toxicity or impurities. Moreover, aspects such as ease of processing, maintenance of sterility, adherence to regulatory approvals, and cost-effectiveness all warrant careful consideration. The biomaterial’s interaction with tissue, its potential for immunogenicity, its long-term stability, and ethical concerns regarding its source and environmental impact all hold pivotal roles in the decision-making process. Tailoring the choice to a specific application and drawing insights from previous research in the field can provide valuable guidance. Ultimately, a rigorous assessment of these critical elements ensures that the selected biomaterial aligns with the goals, safety criteria, and ethical standards of the future study or application.

Finally, we recognize that the lack of quantitative meta-analyses of the articles included limits our evaluation considerably. This is because there were a wide range of reported outcomes, as well as a lack of uniformity in the units or grading systems used. Only one in vitro study was included in this study, resulting in limited findings. The criteria used were justified by the fact that the goal of this study was to highlight the significance of the early stages of biomaterial development. Finally, for future evaluation, statistical analysis is proposed to improve the outcome of this study.

## 5. Conclusions

In conclusion, this study proved that the innovation of biomaterials such as hydrogel, sponge, film 3D-TIPS, and others has made a great contribution to repairing airway epithelia. To adapt to their biological surroundings and to produce a beneficial biological response, the biomaterials must meet several bulk and surface requirements. Moreover, the scaffold design also plays an important role in the development of a quality biomaterial such as the selection of material either from natural or synthetic sources. This selection should be emphasized due to the effect of the material itself on the physicochemical and mechanical properties, topography, and cellular response of biomaterial. Finally, these summary findings provide new information for further investigations, especially in the respiratory system, and at the same time might be more beneficial in the development of new advanced biomaterials to gain better results.

## Figures and Tables

**Table 1:** List of inclusion/exclusion criteria.

Inclusion Criteria	Exclusion Criteria
English language Biomaterial, for example scaffold or hydrogel or others Applied in airway epithelium/respiratory epithelium Studies published between 2012 and 2022	Language other than English Non-full-text original research articles All editorials, conference papers, news, case reports, review papers, and letters

## Data Availability

The data presented in this study are available upon request from the corresponding author.
